# Completion of Multidose COVID-19 Vaccination Among Adolescents and Adults in Urban Informal Settlements in Nairobi, Kenya

**DOI:** 10.4269/ajtmh.24-0737

**Published:** 2025-06-10

**Authors:** Maurine Ng’oda, Jonathan Izudi, Collins Otieno, Daniel Mwanga, Richard E. Sanya, Abdhalah Ziraba

**Affiliations:** ^1^ Emerging and Re-Emerging Infectious Diseases Unit, African Population and Health Research Center, Nairobi, Kenya;; ^2^ Data Science and Evaluation, African Population and Health Research Center, Nairobi, Kenya;; ^3^ Department of Community Health, Faculty of Medicine, Mbarara University of Science and Technology, Mbarara, Uganda

## Abstract

Completion of vaccine doses is essential for robust immunity and long-term protection against specific diseases. This study aimed to investigate factors associated with the completion of multidose coronavirus disease 2019 (COVID-19) vaccines (MDVs) among adolescents and adults in two informal urban settlements in Nairobi, Kenya. We analyzed data from the Kenya Multisite Integrated Serosurveillance project. We defined completion of MDV as receiving an additional COVID-19 vaccine dose between weeks 6 and 8 after the first COVID-19 vaccination shot. We applied the modified Poisson regression model with robust standard errors to determine the factors that were independently associated with the completion of MDV. We analyzed data from 402 individuals aged 14–90 years and found that the completion rate for MDV was 85.3%. In the adjusted analysis, participants aged ≥60 years (adjusted prevalence risk ratio [aPR] 1.30, 95% CI 1.05–1.60) and those who recommended the COVID-19 vaccine to others (aPR 1.40, 95% CI 1.00–1.96) were significantly more likely to complete MDV. Individuals aged 25–59 years (aPR 1.22, 95% CI 0.99–1.50), those who perceived themselves as being at risk for COVID-19 (aPR 1.09, 95% CI 0.99–1.19), and those who had access to healthcare during the pandemic tended to have a higher completion of MDV. Overall, the MDV completion rate is relatively high; however, public health interventions should endeavor to target those being left behind such as younger individuals and those hesitant about vaccination.

## INTRODUCTION

The coronavirus disease 2019 (COVID-19) pandemic posed unprecedented challenges to global public health, with vaccination emerging as one of the most effective tools to mitigate transmission and reduce severe outcomes such as hospitalization and mortality.^1^ By August 12, 2024, 13.72 billion doses of COVID-19 vaccine had been administered globally. However, Africa had received the least number of doses (874.76 million),^2^ revealing significant gaps in both vaccine access and uptake.^3^^,^^4^

In Kenya, the most commonly used COVID-19 vaccines included Oxford/AstraZeneca, Moderna, and Pfizer, and each requires multiple doses to ensure complete immunization.^5^ The completion of multidose vaccine regimens is critical for eliciting stronger immunity and longer-term protection against severe COVID-19. The second dose significantly enhances the immune response, boosting the efficacy to over 90% in preventing severe disease.^6^ Failure to complete multidose regimens undermines this protection and potentially leads to vaccine failure, greater risks for infection, and reduced overall effectiveness of immunization programs including increased susceptibility to COVID-19 variants and reduced community-level immunity.^7^

Disparities in the completion of multidose vaccines for COVID-19 (MDVs) exist, particularly in low-income and marginalized populations such as urban informal settlements.^8^ Urban informal settlements are often characterized by overcrowding, inadequate healthcare infrastructure, and limited access to essential services. In Kenya, an estimated 60% of the residents in Nairobi City reside in informal settlements. The overall COVID-19 vaccine uptake among this population is around 34%,^9^ but the completion of MDV is understudied. Understanding the completion of MDV is crucial for developing targeted interventions that improve COVID-19 vaccine uptake in such settings.

Therefore, we investigated the prevalence of completion of MDV and the associated factors among adolescents and adults in two large, urban, informal settlements in Nairobi, Kenya.

## MATERIALS AND METHODS

### Study setting.

Data used in this paper came from the Kenya Multisite Integrated Serosurveillance (KEMIS) for COVID-19 and Other Pathogens Project. The KEMIS Project was jointly implemented by the African Population and Health Research Center, the Kenya Medical Research Institute Wellcome Trust Research Program, and the U.S Centers for Disease Control and Prevention (CDC). The project aimed to estimate the seroprevalence of severe acute respiratory syndrome coronavirus 2 (SARS-CoV-2) antibodies in the general population. It involved serosurveys conducted among residents of the Kilifi, Nairobi, and Manyatta Health and Demographic Surveillance Systems (HDSS). The details of HDSS have been previously discussed.^9^ The data analyzed in this paper were from the Nairobi site. The Nairobi Urban HDSS (NUHDSS) is established in Nairobi City County, and has consistently tracked a population of approximately 90,000 people each year spread across 30,000 households in two slum areas of Korogocho and Viwandani.^10^

### Study design and eligibility.

Four cross-sectional serosurveys were conducted between October 2020 and June 2024 on a random, age-stratified sample of 850 NUHDSS residents totaling 3,400 observations. These samples consisted of 50 people from each 5-year age group between 15 and 64 years, 50 people aged 65 and above, and 100 children from each 5-year age group between 0 and 14 years.

This resulted in 300 participants under 15 years old sufficient to estimate a 1% outcome measure rate with a 2% margin of error. For the 15–64 age group, 500 participants were deemed adequate to estimate an outcome rate of 3–5% within a 5% margin of error. The survey excluded individuals with bleeding disorders and other medical contraindications for venipuncture or capillary blood sample collection. Data were collected using structured questionnaires and laboratory analysis of blood samples for COVID-19 antibodies. The questionnaire captured sociodemographic information, information on likely exposure to SARS-Cov-2, and clinical information such as COVID-19 symptoms, childhood immunization, death of household members, and vaccination status. Vaccination status was verified using the vaccination certificate or text message confirmation from the national COVID-19 registry. Other information collected included access to health services during the pandemic and loss of income during COVID-19.

### Measurements and variables.

The present study used data from the second and third survey rounds because the first round of the survey took place before the COVID-19 vaccine rollout in the country and the fourth round of the survey did not include key variables that were considered for the current analysis. We considered observations for individuals who were eligible for COVID-19 vaccination and who had received the first dose of MDV, namely AstraZeneca, Moderna, Pfizer, and Sinopharm. We excluded records for the following individuals: those ineligible for the COVID-19 vaccine based on age, who never received any COVID-19 vaccination, who never knew the vaccine they had received at first vaccination, and those who had missing vaccination information.

The primary outcome variable of interest was completion of MDV measured as a binary outcome and defined as the receipt of an additional dose of a COVID-19 vaccine between weeks 6 and 8 after the first dose of an MDV. The independent variables included age measured as the number of years lived and categorized as 14–24, 25–59, and ≥60 years to respectively denote adolescents and young people, middle-aged persons, and older persons; gender (male versus female); religion measured as none, Muslim, Christians, and others; education level measured as none, primary, secondary, and postsecondary; exposure to a suspected COVID-19 case measured as no, yes, probable, and not reported; access to healthcare during the COVID-19 pandemic measured on a binary scale as yes or no; and perceived risk of COVID-19 infection measured as no risk versus at risk. Additionally, we assessed whether the participants were willing to recommend COVID-19 vaccination to others and whether they had any household member who had died of COVID-19.

## STATISTICAL ANALYSES

We summarized categorical variables as frequencies and proportions. Numerical variables were summarized using the mean and SD as they were normally distributed. The χ^2^ test was used to examine differences in MDV completion by the covariates at a 0.15 significance level in the bivariate analysis. We used the modified (robust) Poisson regression model to determine the factors independently associated with MDV completion, adjusting for both clinically and socially relevant variables from the literature, statistically significant variables at the bivariate analysis, and robust standard errors as recommended by Cameron and Trivedi. The unadjusted and adjusted prevalence risk ratios (aPRs) along with the 95% CIs were reported. Variables with *P* <0.05 were considered statistically significant in the multivariable modified Poisson regression analysis.

## RESULTS

### Study profile.

**Figure 1.**
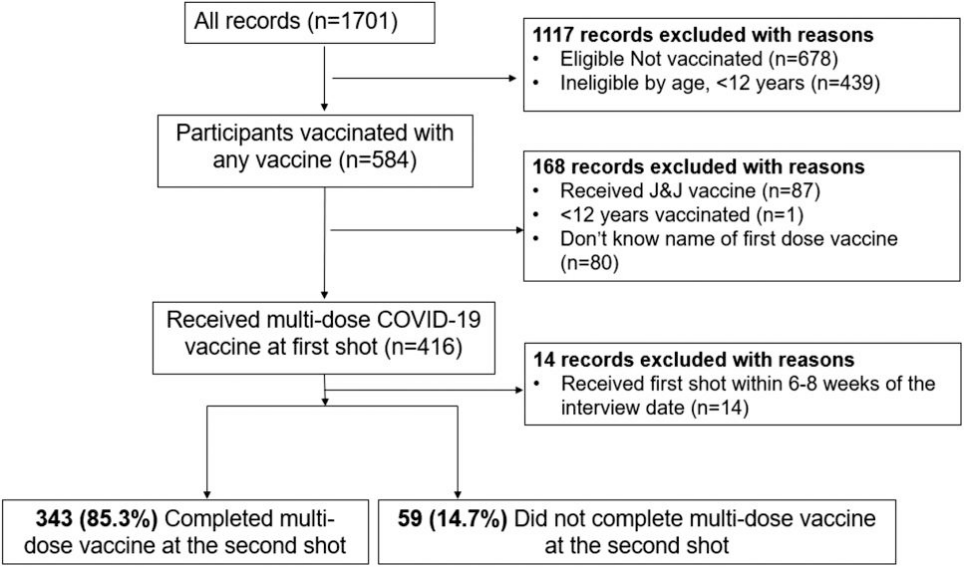
Study profile based on eligibility criteria.

We considered data from 1,701 participants and excluded 1,295 that did not meet the eligibility criteria. Overall, we analyzed 402 participants, of whom 343 (85.0%) completed MDV as shown in Figure 1.

### General characteristics of the participants.

The general characteristics of the participants are presented in Table 1. Of the 402 participants, 262 (65.2%) were from Viwandani study site; 264 (65.7%) were male; 254 (63.2%) were aged 25–59 years and the overall mean age was 48.5 ± 15.7 332 (80.1%) participants who had no known contact with confirmed/suspected COVID-19 cases; 260 (64.7%) perceived themselves at risk for infection; and 361 (89.8%) lacked access to healthcare during the pandemic.

**Table 1:** General characteristics of participants who received multidose COVID-19 vaccine

Variables	Level	All (*n* = 402)
Study site	Korogocho	140 (34.8)
	Viwandani	262 (65.2)
Survey rounds	Round 2	172 (42.8)
	Round 3	230 (57.2)
Age group (years)	14–24	43 (10.7)
	25–59	254 (63.2)
	≥60	105 (26.1)
	Mean (SD)	48.53 (15.71)
Gender	Female	138 (34.3)
	Male	264 (65.7)
Level of education	None	16 (4.0)
	Primary	169 (42.0)
	Secondary	179 (44.5)
	Postsecondary	38 (9.5)
Religion	None	9 (2.2)
	Muslim	13 (3.2)
	Christians	270 (67.2)
	Others	110 (27.4)
Ever exposed to suspected COVID-19 case	No	332 (80.1)
	Yes	36 (9.0)
	Probable	30 (7.5)
	Not reported	14 (3.5)
Had access to healthcare during the COVID-19 pandemic	No	361 (89.8)
	Yes	35 (8.7)
	Not reported	6 (1.5)
Perceived risk of infection with COVID-19	No risk	142 (35.3)
	At risk	260 (64.7)
History of the household member who died of COVID-19	No	370 (92.0)
	Yes	30 (7.5)
	Do not know	2 (0.5)
Would recommend the COVID-19 vaccine to others	No	19 (4.7)
	Yes	383 (95.3)

COVID-19 = coronavirus disease 2019.

### Type and timing of receipt of multidose COVID-19 vaccine (MDV).

The types and timing of MDV at the first and second shots are summarized in Table 2. At the first shot, 266 (66.2%) participants received the AstraZeneca vaccine, 61 (15.2%) received Pfizer, 74 (18.4%) received Moderna, and one (0.2%) received the Sinopharm vaccine. At the second shot, the number of participants who received the AstraZeneca vaccine dropped to 224 (55.7%), and that for Pfizer and Moderna dropped to 59 (14.7%) and 56 (13.9%), respectively. One participant received Sinopharm at the first and second shots. At the second shot, 59 (14.7%) participants did not return for their COVID-19 vaccine. Overall, 343 (85.3%) participants completed their MDV in this study.

**Table 2:** Type and timing of receipt of multidose COVID-19 vaccine (MDV)

Variables	Level	Overall (*n* = 402)
COVID-19 vaccine at the first shot	AstraZeneca	266 (66.2)
	Pfizer	61 (15.2)
	Moderna	74 (18.4)
	Sinopharm	1 (0.2)
COVID-19 vaccine at the second shot	AstraZeneca	224 (55.7)
	Pfizer	59 (14.7)
	J & J	1 (0.2)
	Moderna	56 (13.9)
	Sinopharm	1 (0.2)
	Don’t know	2 (0.5)
	Never returned	59 (14.7)
Completed multidose COVID-19 vaccine	No	59 (14.7)
	Yes	343 (85.3)

COVID-19 = coronavirus disease 2019; MDV = multidose COVID-19 vaccine.

### Completion of MDV by the sociodemographic characteristics and other factors.

The completion of MDV varied across different sociodemographic characteristics and other influencing factors as shown in Table 3. Completion rates were high across both study sites, with 85.7% in Korogocho and 85.1% in Viwandani (*P* = 0.871). Individuals aged ≥60 years had the highest completion rate at 91.4%, whereas those aged 14–24 had a lower completion rate at 69.8% (*P* = 0.003). The mean age of completers was significantly higher (50.14 years) compared with non-completers (39.17 years, *P* <0.001). There were no significant differences in completion rates by gender, with males and females completing at rates of 85.6% and 84.8%, respectively (*P* = 0.825). Educational level showed modest differences, with those having no education showing the highest completion rate (93.8%), though this was not statistically significant (*P* = 0.504). Religion was not a significant factor, though Muslims reported a 100% completion rate compared with 86.7% among Christians (*P* = 0.102).

**Table 3:** Completion of MDV by the sociodemographic characteristics and other factors

Variables	Level	Completed MDV		*P*-Value
		No (*n* = 59)	Yes (*n* = 343)	
Study site	Korogocho	20 (14.3)	120 (85.7)	0.871
	Viwandani	39 (14.9)	223 (85.1)	
Survey rounds	Round 2	26 (15.1)	146 (84.9)	0.829
	Round 3	33 (14.3)	197 (85.7)	
Age group (years)	14–24	13 (30.2)	30 (69.8)	0.003
	25–59	37 (14.6)	217 (85.4)	
	≥60	9 (8.6)	96 (91.4)	
	Mean (SD)	39.17 (15.73)	50.14 (15.16)	<0.001
Gender	Female	21 (15.2)	117 (84.8)	0.825
	Male	38 (14.4)	226 (85.6)	
Level of education	None	1 (6.3)	15 (93.8)	0.504
	Primary	22 (13.0)	147 (87.0)	
	Secondary	31 (17.3)	148 (82.7)	
	Postsecondary	5 (13.2)	33 (86.8)	
Religion	None	3 (33.3)	6 (66.7)	0.102
	Muslim	0 (0.0)	13 (100.0)	
	Christians	36 (13.3)	234 (86.7)	
	Others	20 (18.2)	90 (81.8)	
Ever exposed to suspected COVID-19 case	No	44 (13.7)	278 (86.3)	0.660
	Yes	7 (19.4)	29 (80.6)	
	Probable	6 (20.0)	24 (80.0)	
	Not reported	2 (14.3)	12 (85.7)	
Had access to healthcare during the COVID-19 pandemic	Yes	3 (8.6)	32 (91.4)	0.562
	No	55 (15.2)	306 (84.8)	
	Not reported	1 (16.7)	5 (83.3)	
Perceived risk of infection with COVID-19	No risk	27 (19.0)	115 (81.0)	0.069
	At risk	32 (12.3)	228 (87.7)	
History of the household member who died of COVID-19	No	56 (15.1)	314 (84.9)	0.166
	Yes	2 (6.7)	28 (93.3)	
	Do not know	1 (50.0)	1 (50.0)	
Would recommend the COVID-19 vaccine to others	Yes	52 (13.6)	331 (86.4)	0.005
	No	7 (36.8)	12 (63.2)	

COVID-19 = coronavirus disease 2019; MDV = multidose COVID-19 vaccine.

The perceived risk of COVID-19 infection was marginally associated with completion, as those perceiving themselves at risk had a higher completion rate (87.7%) compared with those perceiving no risk (81.0%, *P* = 0.069). Finally, willingness to recommend the COVID-19 vaccine was significantly associated with completion compared with those unwilling to recommend the vaccine to others (86.4% vs. 63.2%, *P* = 0.005).

### Factors associated with the completion of MDV at the unadjusted and adjusted analyses.

In the unadjusted analysis (Table 4), participants aged 25–59 years (PR 1.22, 95% CI 1.00–1.50) and ≥60 years (PR 1.31, 95% CI 1.07–1.61) were more likely to complete MDV compared with those aged 14–24 years. Being a male compared with a female was not associated with completion of MDV (PR 1.01, 95% CI 0.93–1.10). Education levels, namely primary (PR 0.93, 95% CI 0.81–1.07), secondary (PR 0.88, 95% CI 0.76–1.02), and postsecondary education (PR 0.93, 95% CI 0.78–1.11) showed no significant association with completion of MDV compared with no formal education. Participants who perceived themselves at risk for COVID-19 (PR 1.08, 95% CI 0.99–1.19) and those who reported that they would recommend the vaccine to others (PR 1.37, 95% CI 0.97–1.93) tended to have a higher likelihood of completion of MDV.

**Table 4:** Factors associated with the completion of MDV

Variables	Level	Modified Poisson Regression Analysis	
		Unadjusted Analysis	Adjusted Analysis
		PR (95% CI)	aPR (95% CI)
Site name	Korogocho	1	1
	Viwandani	0.99 (0.91–1.08)	1.02 (0.93–1.11)
Age group (years)	14–24	1	1
	25–59	1.22* (1.00–1.50)	1.22 (0.99–1.50)
	≥60	1.31*** (1.07–1.61)	1.30** (1.05–1.60)
Gender	Female	1	1
	Male	1.01 (0.93–1.10)	1.02 (0.93–1.11)
Level of education	None	1	1
	Primary	0.93 (0.81–1.07)	0.98 (0.85–1.13)
	Secondary	0.88* (0.76–1.02)	0.95 (0.82–1.10)
	Postsecondary	0.93 (0.78–1.11)	1.01 (0.83–1.23)
Ever exposed to suspected COVID-19 case	No	1	1
	Yes	0.93 (0.79–1.10)	0.90 (0.76–1.07)
	Probable	0.93 (0.77–1.11)	0.93 (0.78–1.11)
	Not reported	0.99 (0.80–1.24)	0.98 (0.81–1.18)
Had access to healthcare during the COVID-19 pandemic	No	1	1
	Yes	1.08 (0.97–1.20)	1.09 (0.97–1.22)
	Not reported	0.98 (0.69–1.41)	1.01 (0.68–1.51)
Perceived risk of infection with COVID-19	No risk	1	1
	At risk	1.08 (0.99–1.19)	1.09 (0.99–1.19)
Would recommend the COVID-19 vaccine to others	No	1	1
	Yes	1.37* (0.97–1.93)	1.40** (1.00–1.96)

aPR = adjusted prevalence risk ratio; COVID-19 = coronavirus disease 2019; MDV = multidose COVID-19 vaccine; PR = prevalence risk ratio. Exponentiated coefficients are the prevalence risk ratios at a 5% significance level; 95% CIs in parentheses: * *P* <0.05, ** *P* <0.01, *** *P* <0.001. Analysis adjusted for the survey round.

In the adjusted analysis (Table 4), participants aged ≥60 years (aPR 1.30, 95% CI 1.05–1.60) and those who reported that they would recommend the COVID-19 vaccine to others (aPR 1.40, 95% CI 1.00–1.96) were more likely to complete MDV. We found a borderline statistically significant association between being aged 25–59 years compared with 14–24 years (aPR 1.22, 95% CI 0.99–1.50), access to healthcare during the COVID-19 pandemic (aPR 1.09, 95% CI 0.97–1.22), and perceived risk of COVID-19 (aPR 1.09, 95% CI 0.99–1.19).

## DISCUSSION

We assessed the factors associated with the completion of MDV in Nairobi’s informal settlements. Our findings indicated that eight in every 10 individuals in the informal settlements who had received a COVID-19 vaccine completed their MDV. Completion of MDV was more likely for middle-aged (25–59 years) and older adults (60+ years) compared with younger participants (14–24 years) and among those reporting willingness to recommend COVID-19 vaccination to others. Furthermore, individuals who perceived themselves at risk for COVID-19 and those who had access to health services during the pandemic tended to have a higher likelihood of completing their MDV.

Age has been shown in previous studies to be a significant determinant of vaccine uptake^11^^,^^12^ and our findings align with this evidence. The high completion rate of MDV among middle-aged individuals (the majority of the working population) may have been influenced by the COVID-19 restrictions that largely affected city employees. Across urban areas, including Nairobi, several employers mandated COVID-19 vaccination as a requirement for continued employment, especially in sectors involving close contact with others, like hospitality, transport, and retail.^13^^,^^14^ These mandates might have incentivized middle-aged workers to complete their MDV regimens to retain their jobs and avoid financial instability. Compared with younger persons, middle-aged and older individuals might have prioritized vaccination to reduce the risk of transmission of SARS-COV-2 (the virus causing COVID-19) at the household level.

Diesel and colleagues (2021) argued that older adults are more likely to complete their COVID-19 vaccine regimens because they are more aware of the health risks associated with COVID-19.^15^ A previous study indicated that older adults prioritize vaccination because of their higher perceived risk of severe outcomes associated with COVID-19.^16^

Participants who reported that they would recommend the COVID-19 vaccine to others were more likely to complete their MDV compared with those who reported otherwise. This finding highlights the critical role of vaccine confidence in shaping health behavior. Vaccine-non-hesitant individuals have been found to have a higher level of trust in the safety and efficacy of vaccines, which motivates them to get vaccinated and advocate for others to equally get vaccinated.^17^ This positive attitude toward vaccination likely translates into greater adherence to completing the full vaccine schedule because such individuals likely appreciate the benefits of vaccination. Contrastingly, vaccine-hesitant individuals may harbor doubts about vaccine safety or question its necessity and hence are less likely to commit to completing the multidose regimens.^17^ The potential reasons for not completing MDV include perceived lack of need for vaccination, cultural influences, demographic factors such as younger age and lower education levels, mistrust of vaccine manufacturers, misinformation, conflicting/confusing media messages, perceived good health, and the presence of comorbidities. Some of these reasons have been reported in previous studies.^18^^–^^21^ This finding emphasizes the importance of addressing vaccine hesitancy through public health messaging and community engagement, particularly in settings like informal urban settlements where misinformation and distrust are prevalent.

Individuals with a perceived risk of COVID-19 infection showed a marginally higher tendency to complete their MDV. We argue that feeling at risk of COVID-19 infection, by itself, may not sufficiently motivate an individual to adhere to vaccination schedules, but rather other factors such as the perceived benefits of full vaccination, easy access to vaccines, trust in the health system, and influences from social networks.^22^^–^^24^ Even though some individuals might acknowledge that they are at risk for COVID-19, they may still hesitate to get vaccinated because of concerns about vaccine side effects, doubts about its effectiveness, or general mistrust of health interventions.^24^^,^^25^ Moreover, the lack of significant findings might suggest that people often need more than just a sense of risk to act. Public health theories such as the Health Belief Model highlight that, although perceived risk can increase awareness, it often takes “cues to action” to trigger a response.^26^ These cues could include reminders from health professionals, public health campaigns, or healthy public policies such as vaccine mandates.

There are also other important dynamics at play such as vaccine fatigue.^27^ Some people might have been eager to get their initial dose but then become reluctant to receive subsequent doses because of misinformation or concerns after the first vaccination. This highlights that even when perceived risk is high, other competing factors may prevent people from completing their vaccination schedule.

Structural barriers such as limited access to vaccines, financial constraints, or the inability to take time off work also negatively affect people in urban slum environments regarding vaccination uptake. In urban informal settlements, basic needs such as food or income are prioritized over long-term health needs such as vaccination.

Overall, public health strategies that focus exclusively on raising awareness of the risks associated with COVID-19 may not be effective in improving vaccine completion.

This study has numerous strengths and some limitations. The use of a modified Poisson regression analysis prevented the overestimation of associations between the independent variables and the outcome, hence providing better estimates. Missing data, particularly from participants who were unaware of the type of vaccine they had received at the first shot, might have led to either under or overestimation of the completion of MDV. The data analyzed were collected through self-reporting, presenting a possible social desirability bias. Findings are from an urban informal settlement so may not generalize to other settings. Despite these limitations, the study provides credible findings about the completion of MDV in urban informal settlements where access to healthcare remains problematic and individuals are at a higher risk for diseases, complications, and mortality.

## CONCLUSION

We conclude that the completion rate for MDV is relatively high. However, to further enhance vaccination coverage, public health interventions should focus on targeted education, emphasizing the importance of completing the full vaccination schedule. These efforts should prioritize younger individuals and those displaying vaccine hesitancy, ensuring that critical gaps in coverage are addressed and sustained immunity is achieved within the population.
